# Relation of Secondhand Smoking to Mild Cognitive Impairment in Older Inpatients

**DOI:** 10.1100/2012/726948

**Published:** 2012-05-01

**Authors:** Giuseppe Orsitto, Vincenzo Turi, Amedeo Venezia, Francesco Fulvio, Cosimo Manca

**Affiliations:** Geriatric Unit, “Paradiso” Hospital, Azienda Sanitaria Locale Bari, 70023 Gioia del Colle, Italy

## Abstract

Up to now, controversy still exists regarding the role of secondhand smoking (SHS) in developing cognitive impairment. This study aimed to evaluate the prevalence of SHS in hospitalized older patients with cognitive deficit, particularly in those with mild cognitive impairment (MCI). Smoking history was classified into four groups: never smokers, former-active smokers/no SHS, active smokers, and secondhand smokers, and cognitive function into three levels: normal cognition (C), MCI, and dementia. A total of 933 older subjects with diagnoses of MCI (*n* = 98), dementia (*n* = 124), or C (*n* = 711) were enrolled in this cross-sectional study. As expected, patients with dementia had significantly higher frequency of former-active smokers than cognitively normal. Moreover, patients with MCI showed a significantly higher frequency of active and secondhand smokers than patients with dementia or C. A smoking history is very frequent in older patients with dementia. Patients with MCI had even higher rate of exposure to active or secondhand smoking.

## 1. Introduction

Recent data clearly confirm that active smoking in older people is a significant risk factor for dementia [1–3], including Alzheimer's Disease (AD) [4], although the scientific literature has reported conflicting results about this topic, with early reports suggesting no significant association between smoking and cognitive impairment [5, 6] or surprisingly its protective action against dementia [7–9]. There is also growing evidence that exposure to secondhand smoking (SHS) in older nonsmokers may be associated with increased odds of dementia [10], although remains still unclear the relationship between SHS and the preclinical phase of dementia, particularly mild cognitive impairment (MCI), in older patients. MCI is a transitional and heterogeneous clinical syndrome that lies between normal aging and early dementia which refers to nondemented, aged persons with memory or cognitive impairment and no significant disability [11, 12]. In the developed world, the prevalence of MCI is more than double that of dementia and its rate of conversion to dementia is greater than that of general population.

The aim of this study was to evaluate the smoking history including the prevalence of exposure to SHS in hospitalized older patients with cognitive deficit, particularly in those with MCI.

## 2. Methods

### 2.1. Participants

The study population included all patients aged 65 and older consecutively admitted to the geriatric ward of the “Paradiso” Hospital, Azienda Sanitaria Locale Bari (ASL BA), Gioia del Colle (Ba), Italy, from January 2010 to July 2011. Written informed consent was obtained from all patients or from relatives of critically ill or demented patients prior to participation in the study.

### 2.2. Study Evaluation

At admission, a comprehensive geriatric assessment (CGA) [13], including basic and instrumental activities of daily living (ADLs, IADLs) [14, 15], cumulative illness rating scale comorbidity index (CIRS severity and CIRS comorbidity) [16], mini-mental state examination (MMSE) [17], clinical dementia rating scale (CDR) [18], geriatric depression scale 15-item (GDS-15) [19], and mini-nutritional assessment (MNA) [20], was performed by the staff physician at the ward. The smoking history, evaluated by a structured questionnaire self-reported by patients or obtained from relatives of critically ill or demented patients, was classified into four groups: never smokers, former-active smokers/no SHS, active smokers, and secondhand smokers. “Never smokers” were defined as those who had not smoked more than 100 cigarettes in their lifetime, “former-active smokers/no SHS” as those who had stopped smoking for at least two years at the date of the interview, “active smokers” as having smoked a total of at least twenty packs of cigarettes over their lifetime and at least one cigarette per day in the last year. “Secondhand smokers” were finally defined as those who had at least one hour/week exposure to SHS. SHS among never or former-active smokers was assessed ascertaining exposure in seven microenvironments: the respondent's home, another person's home, travelling in a car or another vehicle, workplace, bars, outdoor locations, and other locations. In each area, was evaluated the total duration (in hours) of exposure during the past seven days.

The cognitive function was categorized into three levels—MCI, dementia, or normal cognition (NoCI)—according to the neuropsychological test score, as detailed elsewhere [21].

Diagnosis of MCI was made using the following Petersen criteria [11, 12]: presence of subjective memory loss, preferably corroborated by an informant; demonstration of a memory impairment by cognitive testing; preserved general intellectual functioning as estimated by performance on a vocabulary test; intact ability to perform activities of daily living and absence of dementia. Diagnoses of possible/probable Alzheimer's disease (AD), vascular dementia (VaD), and mixed dementia (MD) were made according to the criteria of the National Institute of Neurological and Communicative Disorders and Stroke/Alzheimer's Disease and Related Disorders Association Work Group [22], the National Institute of Neurological Disorders and Stroke-Association Internationale pour la Recherche et l'Enseignement en Neurosciences Work Group [23], and the Diagnostic and Statistical Manual of Mental Disorders—Fourth Edition (DSM-IV) [24].

In agreement with the exclusion criteria for MCI defined by Levy [25], subjects who had present or past medical or psychiatric condition or psychoactive substance use that can cause cerebral dysfunction were excluded from the study to rule out the possibility of cognitive impairment due to medical or psychiatric conditions.

### 2.3. Statistical Analyses

Statistical analysis was performed using the SPSS V. 11.5 for Windows statistical software package. Pearson's *χ* ^2^ test and Fisher's exact test were used to compare frequencies of patients according to their smoking history. The Kruskal-Wallis test was applied to compare demographic, cognitive, functional, and nutritional characteristics as well as comorbidity of the study samples. Logistic regression analysis adjusted for age, educational level, and never smokers was also used to confirm the association between patients smoking history and cognitive impairment. The ANOVA post hoc analysis was used for correction in multiple comparisons. All *P* values were 2-tailed, with statistical significance indicated by a value of *P* < 0.05.

## 3. Results

During the study period, 1128 subjects aged 65 and older were admitted to the hospital's geriatric ward and were screened. In 63 subjects, data were not recorded, and 97 patients were excluded from the study because of a diagnosis of short-term prognosis tumors (*n* = 20), blood infections (*n* = 18), serious anemia (*n* = 15), disorders of the thyroid (*n* = 13), primary or secondary malignant brain neoplasms (*n* = 12), alcohol abuse (*n* = 10), head trauma (*n* = 6), and hydrocephalus (*n* = 3). Thirty-five patients refused to participate in the study. Patients who had stopped smoking during last two years (*n* = 36) were also excluded from the study, but all of them presented one of the several above-mentioned exclusion criteria or refused to participate in the study. Thus, the final analysis included data from 933 older subjects (394 men, 539 woman, mean age 77.2 ± 6.9, range 65–102 years): 124 patients with dementia (AD = 37 patients, VaD = 45, MD = 42), 98 patients with MCI, and 711 cognitively normal.

Table 1 shows the mean results of variables, expressed according to the cognitive levels. Patients with dementia had significantly lower MMSE scores (*P* < 0.001), educational level (*P* < 0.001), and MNA scores (*P* < 0.05) and higher CDR scores (*P* < 0.001), mean age (*P* < 0.05), and level of disability (*P* < 0.0001) than patients with MCI or C. No significant differences were found in CIRS severity, CIRS comorbidity, and GDS scores between the three groups. Moreover, MCI patients had significantly lower MMSE and higher CDR scores (*P* < 0.001) than C, but no significant differences were found in the level of education, mean age, level of disability, and MNA scores between the two groups. Also no significant differences were found among patients with various dementia diagnoses (i.e., AD, VaD, or MD) with regard to the above-mentioned variables.

Table 2 illustrates the distribution of the exposure to cigarette smoking according to the cognitive levels of the sample study. As expected, the frequency of never smokers in the patients without cognitive deficit was significantly higher than that in the patients with dementia and MCI (*P* < 0.0001; OR = 3.9, CI = 2.1–8.3). However, the frequency of former-active smokers/no SHS in patients with dementia was significantly higher than that in C (*P* < 0.0001; OR = 2.5, CI = 1.1–5.2), but no significant differences were found between patients with dementia and MCI (*P* = 0.07; OR = 1.0, CI = 0.5–1.4). Moreover, patients with MCI showed a significantly higher frequency of active smokers that patients with dementia (*P* < 0.005; OR = 2.3, CI = 1.4–4.5) and C (*P* = 0.04; OR = 3.0, CI = 1.4–5.9), but no significant differences were found between patients with dementia and C (*P* = 0.09; OR = 1.0, CI = 0.5–1.6). Similarly, the frequency of the secondhand smokers was significantly higher in the patients with MCI than that in the patients with dementia (*P* < 0.03; OR = 2.7, CI = 1.5–4.4) or C (*P* < 0.0001; OR = 1.9, CI = 1.0–3.5), but no significant differences were found between patients with dementia and C (*P* = 0.19; OR = 0.9, CI = 0.4–1.5). At last, no significant differences were found in frequencies of never smokers, former-active smokers/no SHS, active smokers, and secondhand smokers among patients with various above-mentioned dementia diagnoses.

## 4. Discussion

Clinical research has recently focused on the identification and characterization of early markers of dementia, in order to identify patients at high risk and to implement preventive measures to delay the onset of disease. Currently, there is an increased interest in modifiable risk factors determining progression to dementia such as cigarette smoking. Several studies have well demonstrated that active smoking may be a risk factor for dementia in older people [1–4], although other studies have shown no significant association [5, 6] or surprisingly a protective action of cigarette smoking against dementia [7–9], probably due to the protective effect of nicotine at least in the short term [26]. However, controversy still exists particularly concerning the association between secondhand smoking and the preclinical phase of dementia, including MCI. The lack of knowledge about this topic may be due to several reasons, including the difficulties in objectively quantifying exposure to tobacco using large-scale analysis methods, varying stringency in the diagnostic criteria used in particularly in MCI, and the limited sample size of frail older people enrolled in clinical trials. So, in the present study, we evaluated the relationship between exposure to cigarette smoking and different grades of cognitive impairment, particularly MCI, in a sample of hospitalized older patients from the Apulia region of southern Italy. Surprisingly, we described an extremely high prevalence of smoking history in our sample study of hospitalized older patients: only one-third of these patients were classified as never smokers, while remaining of them reported a current or past history of cigarette smoking. In agreement with recent studies [1–4] that showed a relationship between cigarette smoking and deep cognitive impairment, our data confirmed a significantly higher percentage of smoking history in patients with dementia than those without cognitive impairment. However, the major finding of this study is undoubtedly represented by the evidence of significantly higher percentages of active smokers in patients with MCI who are the ones at higher risk of progression to dementia. This finding is also supported by evidence of significantly higher percentages of secondhand smokers in patients with MCI, and is consistent with data recently reported by Barnes et al. [10]. This evidence, that according to literature reviewed is the first found in a cohort of older inpatients from Apulia, is of potential clinical value and might suggest that cigarette smoking plays a role in the transitional process from normal cognition to mild cognitive disorders, as recently reported [27] and therefore that further efforts to ensure smoking cessation and prevention programs toward older people are necessary. Longitudinal studies are particularly needed to further investigate and confirm the exact role of secondhand smoking in developing cognitive impairment in nondemented older people, possibly supported by innovative methods such as the analysis of salivary concentrations of cotinine, a metabolite of nicotine recently used as biomarker for recent exposure to secondhand smoking [28]. In line with the findings of Anstey et al. [2], our data also indicated that the smoking history did not vary in patients with various dementia diagnoses, that is, AD, VaD, or MD. As recently shown [29], this study gave indication of a possible association between dementia and older mean age, greater functional disability, and lower educational level and nutritional status. Comparing patients with dementia according to diagnosis, no significant differences were found in mean age, educational level, depression scores, or level of disability.

## 5. Limitations

This study has several limitations. First, its cross-sectional design prevents any conclusion about the chronology and the causality of the observed relationship between smoking history and cognitive decline that can be only supposed. Second, results of our sample study of hospitalized older patients cannot be extended to general population. Lastly, the self-report questionnaire used, as previously mentioned, represents an insufficient method to analyze and objectively quantify exposure to tobacco.

## 6. Conclusions

In conclusion, the results of the present study showed a high prevalence of smoking history in hospitalized elderly patients with dementia. However, the major finding in this population of older people from southern Italy was the evidence of a higher rate of exposure to active or secondhand smoking in patients with MCI who had not progressed to dementia, compared to those with no cognitive decline. This novel finding suggested that there may be benefit in smoking cessation and prevention programs toward older people particularly in the MCI group. Further intervention studies including innovative methods that are able to objectively quantify exposure to tobacco will undoubtedly be informative in this regard.

## Figures and Tables

**Table 1:** Demographic and clinical characteristics of patients according to the cognitive levels.

	Dementia (*n* = 124)	MCI (*n* = 98)	C (*n* = 711)
Age (years)	81.0 ± 6.2^∗‡^	76.9 ± 6.5	75.5 ± 6.9
Gender (female)	83 (69.9%)	65 (66.3%)	391 (54.9%)
Instruction (years)	3.2 ± 3.7^†‡^	5.2 ± 4.0	5.6 ± 2.9
Mini mental state examination	16.2 ± 5.0^†‡^	24.4 ± 1.5^§^	27.0 ± 1.8
Clinical dementia rating scale	1.4 ± 1.1^†‡^	0.5^§^	0
Activities of daily living	3.5 ± 2.0^†‡^	5.1 ± 1.1	5.3 ± 1.0
Instrumental activities of daily living	2.5 ± 2.1^†‡^	5.9 ± 2.3	6.2 ± 2.2
Cumulative illness rating scale severity	1.8 ± 0.4	1.6 ± 0.5	1.5 ± 0.7
Cumulative illness rating scale comorbidity	2.5 ± 1.5	2.4 ± 1.7	2.7 ± 1.5
Geriatric depression scale	6.0 ± 4.4	5.1 ± 3.1	5.7 ± 3.0
Mini-nutritional assessment	18.3 ± 4.7^∗‡^	20.4 ± 4.0	20.9 ± 3.5

Mean ± standard deviation and frequency (%) are reported. The ANOVA post hoc for multiple comparison analysis confirmed the statistical significance of the *P* values.

*Dementia versus MCI *P* < 0.05; ^†^Dementia versus MCI *P* < 0.001; ^‡^Dementia versus C *P* < 0.001; ^§^MCI versus C *P* < 0.001.

**Table 2:** Cognitive levels and smoking history.

	Dementia (*n* = 124)	MCI (*n* = 98)	C (*n* = 711)	Total (*n* = 933)
Never smokers	25 (20)^∗†^	9 (9)^‡^	306 (43)	340 (36.5)
Former-active smokers/no SHS	79 (64)*	49 (51)	284 (40)	412 (44.5)
Active smokers	5 (4)^†^	16 (16)^§^	64 (9)	85 (9)
Secondhand smokers	15 (12)^†^	24 (24)^‡^	57 (8)	96 (10)

*χ* ^2^ test

Number of patients and frequency (%) are reported.

*Dementia versus C *P* < 0.001; ^†^Dementia versus MCI *P* < 0.05; ^‡^MCI versus C *P* < 0.001; ^§^MCI versus C *P* < 0.05.
